# ﻿Description of two new species of *Dicranomyia* (*Erostrata*) crane fly (Diptera, Limoniidae) from Korea, with remarks on DNA barcoding and updated taxonomic key

**DOI:** 10.3897/zookeys.1157.90792

**Published:** 2023-04-11

**Authors:** Jisoo Kim, Yeon Jae Bae

**Affiliations:** 1 Department of Environmental Science and Ecological Engineering, Graduate School, Korea University, Seoul 02841, Republic of Korea Korea University Seoul Republic of Korea

**Keywords:** Dicranomyia (Erostrata) jejuensis, Dicranomyia (Erostrata) koreana, DNA barcode, Limoniinae, taxonomy

## Abstract

Two new crane fly species, Dicranomyia (Erostrata) jejuensis**sp. nov.** and D. (E.) koreana**sp. nov.**, from Korea are described on the basis of morphology and mitochondrial *COI* sequences. DNA barcode sequences for other four D. (Erostrata) species from Korea are also provided for the first time. The identification key for all known D. (Erostrata) species is presented.

## ﻿Introduction

Genus *Dicranomyia* Stephens, 1829, is the largest genus of the Limoniidae and, as such, contains 1,136 species and 24 subgenera, including the subgenus D. (Erostrata) Savchenko, 1976. Twelve species of this subgenus have been reported from the Palearctic, Nearctic, and Oriental regions (Oosterbroek 2023). Adult crane flies are typically found in moist deciduous forests, shrubs along small streams, and abandoned farmlands near streams (Alexander 1931a, b; Podenas et al. 2019, 2020). Meanwhile, larval D. (E.) globithorax has been reported in fungus on decaying logs (Rogers 1930). Data on DNA barcoding for this subgenus is unknown so far.

Alexander (1934) first reported D. (E.) tabashii (as Limonia (Limonia) tabashii) from Suigen (= Suwon) in Korea, and Podenas et al. (2019, 2020) added three and one additional species, respectively, to the recognized fauna of Korea. In Japan, Kato et al. (2018) revised six Dicranomyia species of the subgenus Erostrata including three new species and suggested that *Limoniacongesta* Alexander, 1976 and *Limoniastriopleura* Edwards, 1919 be classified as members of subgenus Erostrata based on non-genitalic characters.

Here, two new D. (Erostrata) crane fly species are described from Korea, providing identification for all Korean members of the subgenus. A DNA barcode (*COI*) dataset for six Dicranomyia species of the subgenus Erostrata from Korea is also presented for the first time.

## ﻿Materials and methods

### ﻿Crane fly sampling and examination

Crane fly adults were collected using insect nets or Malaise traps and preserved in 80% ethanol (Table 1). Wings and legs of selected adults were slide mounted using Euparal. Meanwhile, the genitalia and ovipositors of male and female specimens, respectively, were cleared overnight using 10% KOH and then preserved in micro-vials with glycerol. Specimens were examined using a microscope Olympus SZ51 with a digital camera Canon EOS 6D (Tokyo, Japan) and Olympus BX53 with camera Nikon Z7 (Tokyo, Japan).

**Table 1.** Collection data of the Korean Dicranomyia (Erostrata) species used in the barcode analyses of this study.

Species*	GenBank accession number	Specimen code	Locality	Date	Collector(s)	Coordinates
D. (E.) globithorax	OM102980	CF21-0149	Gangwon-do, Wonju-si	3 Sep. 2021	J. Kim, D. Lee	37°29'50.88"N, 130°53'23.78"E
D. (E.) jejuensis sp. nov.	OM102981	CF21-0150H	Jeju-do, Seoguipo-si	14 Jun.–4 Aug. 2021	Y. J. Bae	33°19'49.07"N, 126°37'28.08"E
	OM102982	CF21-0150P	Jeju-do, Seoguipo-si	4 Aug.–8 Sep. 2021	Y. J. Bae	33°19'49.07"N, 126°37'28.08"E
	OM102983	CF21-0151	Jeju-do, Seoguipo-si	4 Aug.–8 Sep. 2021	Y. J. Bae	33°19'49.07"N, 126°37'28.08"E
D. (E.) koreana sp. nov.	OM102979	CF21-0148	Jeju-do, Seoguipo-si	13 Jun.–4 Aug. 2021	Y. J. Bae	33°20'57.10"N, 126°29'43.29"E
	OP081140	CF21-0152	Gyeongsangnam-do, Sancheong-gun	28 Jul. 2021	J. Kim, C. Lim, D. Lee, W. Lee	35°18'37.83"N, 127°45'05.47"E
D. (E.) submelas	OM102978	CF21-0133	Jeju-do, Seoguipo-si	4 Aug.–8 Sep. 2021	Y. J. Bae	33°19'49.07"N, 126°37'28.08"E
D. (E.) tabashii	OM102975	CF21-0115	Gyeongsangnam-do, Sancheong-gun	28 Jul. 2021	J. Kim, C. Lim, D. Lee, W. Lee	35°18'37.83"N, 127°45'05.47"E
	OM102976	CF21-0115f	Gyeongsangnam-do, Sancheong-gun	28 Jul. 2021	J. Kim, C. Lim, D. Lee, W. Lee	35°18'37.83"N, 127°45'05.47"E
D. (E.) yazuensis	OM102977	CF21-0132	Gangwon-do, Pyeongchang-gun	28 Jul.–15 Sep. 2020	Y. J. Bae	37°47'05.67"N, 128°34'16.97"E
D. (D.) kandybinae	OP093621	CF21-0099	Gangwon-do, Wonju-si	23 Jul.–3 Sep. 2021	Y. J. Bae	37°17'26.50"N, 128°04'54.77"E

*Specimens were morphologically identified by J. Kim.

The terminologies used to describe the morphology generally follow Cumming and Wood (2017), and de Jong (2017) for wing venation. The species distribution is given according to Oosterbroek (2023).

Specimen depositories are as follows: **KUEM** – Korea University Entomological Museum, Seoul, Republic of Korea; **NIBR** – National Institute of Biological Resources, Incheon, Republic of Korea.

### ﻿DNA extraction and sequence generation

Total genomic DNA was extracted from the leg muscle of using the DNeasy Blood & Tissue Kit (Qiagen, Hilden, Germany) according to the manufacturer’s instructions. *COI* sequences were amplified and sequenced following Suh et al. (2019), except for the use of primers LCO1490 (5'-GGT CAA CAA ATC ATA AAG ATA TTG G-3'; Folmer et al. 1994) and C1-N-2191 (5'-CCC GGT AAA ATT AAA ATA TAA ACT TC-3'; Simon et al. 1994), which targeted a 676-bp region of *COI*. All sequences were submitted to GenBank (accession numbers: OM102975–OM102983; OP081140; OP093621).

### ﻿DNA barcode sequence analysis

DNA barcode analysis was performed using 11 *COI* sequences (Table 1), which were generated from six Korean D. (Erostrata) species (10 sequences), and the outgroup species D. (Dicranomyia) kandybinae Savchenko, 1987 (1 sequence). Phylogenetic analyses were conducted using the neighbor-joining (NJ) method and Kimura-2-parameter model (Kimura 1980), with 1,000 bootstrap replicates, in MEGA X (Kumar et al. 2018). Sequence divergence was estimated via pairwise comparison of the uncorrected genetic distances (*p*-distances) in MEGA X, using the complete deletion option.

## ﻿Checklist of the world Dicranomyia (Erostrata) crane flies (Oosterbroek, 2023)

Dicranomyia (Erostrata) canis (Alexander, 1931b)

Dicranomyia (Erostrata) cnephosa (Alexander, 1959)

Dicranomyia (Erostrata) congesta (Alexander, 1967)

Dicranomyia (Erostrata) cynotis (Alexander, 1931a)

Dicranomyia (Erostrata) globithorax Osten Sacken, 1869

Dicranomyia (Erostrata) globulithorax Alexander, 1924

Dicranomyia (Erostrata) jejuensis sp. nov.

Dicranomyia (Erostrata) koreana sp. nov.

Dicranomyia (Erostrata) melas (Alexander, 1934)

Dicranomyia (Erostrata) reniformis Kato, Tachi & Gelhaus, 2018

Dicranomyia (Erostrata) striopleura (Edwards, 1919)

Dicranomyia (Erostrata) submelas Kato, Tachi & Gelhaus, 2018

Dicranomyia (Erostrata) tabashii (Alexander, 1934)

Dicranomyia (Erostrata) yazuensis Kato, Tachi & Gelhaus, 2018

### ﻿Key to the species of Dicranomyia (Erostrata), updated from Kato et al. (2018) and Podenas et al. (2020)

**Table:** 

1	Tarsi with white bands	**Dicranomyia (Erostrata) congesta (India)**
–	Tarsi without bands	**2**
2	Pleuron with broad, blackish brown lateral stripe	**Dicranomyia (Erostrata) striopleura (Indonesia, Malaysia)**
–	Pleuron without lateral stripe	**3**
3	Scutellum obscure yellow	**4**
–	Scutellum yellowish brown to blackish brown	**6**
4	Rostrum black. Wing strongly blackened	**Dicranomyia (Erostrata) cnephosa (Nepal)**
–	Rostrum pale. Wing tinged with pale brown	**5**
5	Palpus 2-segmented. Male seventh sternite with strongly darkened internal sac	**Dicranomyia (Erostrata) tabashii (Japan, Korea, Russia)**
–	Palpus 3-segmented. Male seventh sternite with slightly darkened internal sac with rounded entrance	**Dicranomyia (Erostrata) koreana sp.nov. (Korea)**
6	Gonostylus with black spines on mesal face	**7**
–	Gonostylus without black spines	**12**
7	Gonostylus narrowed to a point, triangular	**8**
–	Gonostylus not as above	**9**
8	Mesal face of gonostylus densely covered with black setae	**Dicranomyia (Erostrata) cynotis (Philippines)**
–	Mesal face of gonostylus with black setae restricted to distal 1/2	**Dicranomyia (Erostrata) canis (Philippines)**
9	Paramere distally with rounded tip	**10**
–	Paramere distally with pointed tip	**11**
10	Gonostylus elongate with truncated apex	**Dicranomyia (Erostrata) globithorax (Canada, Japan, Korea, USA)**
–	Gonostylus elongate with angled apex, length of angled apex ca 1/5 of gonostylus	**Dicranomyia (Erostrata) globulithorax (Japan, Korea, Russia)**
11	Gonocoxite with apically rounded ventromesal lobe. Gonostylus shallowly concaved at inner apical edge, the area 1/5 as long as gonostylus	**Dicranomyia (Erostrata) melas (Taiwan)**
–	Gonocoxite with apically truncated ventromesal lobe. Gonostylus deeply emarginated at inner apical edge, the area 1/3 as long as gonostylus	**Dicranomyia (Erostrata) submelas (Japan, Korea)**
12	Gonostylus stout and reniform, tip of ventral surface covered with dense, fine setae	**Dicranomyia (Erostrata) reniformis (Japan)**
–	Gonostylus not reniform, tip of ventral surface without dense, fine setae	**13**
13	Palpus 2-segmented. Gonostylus tapered in distal 1/3, without apical spine	**Dicranomyia (Erostrata) yazuensis (Japan, Korea)**
–	Palpus 3-segmented. Gonostylus tapered in distal 1/2, with black apical spine	**Dicranomyia (Erostrata) jejuensis sp. nov. (Korea)**

### ﻿Taxonomic accounts

**Family Limoniidae Speiser, 1909**

**Subfamily Limoniinae Speiser, 1909**

#### Genus *Dicranomyia* Stephens, 1829

##### ﻿Subgenus Erostrata

Taxon classificationAnimaliaDipteraLimoniidae

Savchenko, 1976

C69BCB6D-4C3F-5FAE-A61B-87A541D8AD53

Dicranomyia (Erostrata) Savchenko in Savchenko and Krivolutskaya 1976: 131–132; Kato et al. 2018: 182; Podenas et al. 2019: 72–73.

###### Type species.

*Dicranomyiaglobithorax* Osten Sacken, 1869 (original designation).

###### Diagnosis.

Rostrum is very short or reduced. Number of palpomeres ranges from one to three. Wings have no patterns, even in stigmal region. Third and fourth tarsomere are slightly swollen. Internal sac or notch is located on the male seventh sternite. Gonocoxite has ventromesal lobe. Gonostylus is one paired, with one or two setae arising from small tubercle on outer surface.

##### ﻿ Dicranomyia (Erostrata) jejuensis sp. nov.

Taxon classificationAnimaliaDipteraLimoniidae

15076BEE-3A44-562E-8FC1-6CF43A4FF557

https://zoobank.org/9659D5F8-C578-4FAA-B16A-6AF8A9AE39E5

Figs 1 , 2

###### Type material.

***Holotype***: Korea • ♂; Jeju-do, Seogwipo-si, Namwon-eup, Sillye-ri, Iseungi-oreum Volcanic Cone; 33°20.24'N, 126°37.25'E; alt. 450 m; 4 Aug.–8 Sep. 2021; Y. J. Bae leg.; Malaise trap; GenBank: OM102981; CF21-0150H; NIBR.

***Paratypes***: Korea • 1 ♀; same data as holotype, 14 Jul.–4 Aug. 2021; GenBank: OM102983; CF21-0151; KUEM • 1 ♂; same data as holotype; GenBank: OM102982; CF21-0150P; KUEM.

###### Diagnosis.

Palpus is 3-segmented. Male seventh sternite has shallow V-shaped notch. Outer face of gonostylus has single seta arising from tubercle. Distal lobe of paramere has a hooked tip with a subapical process.

###### Description.

**Figure 1.**
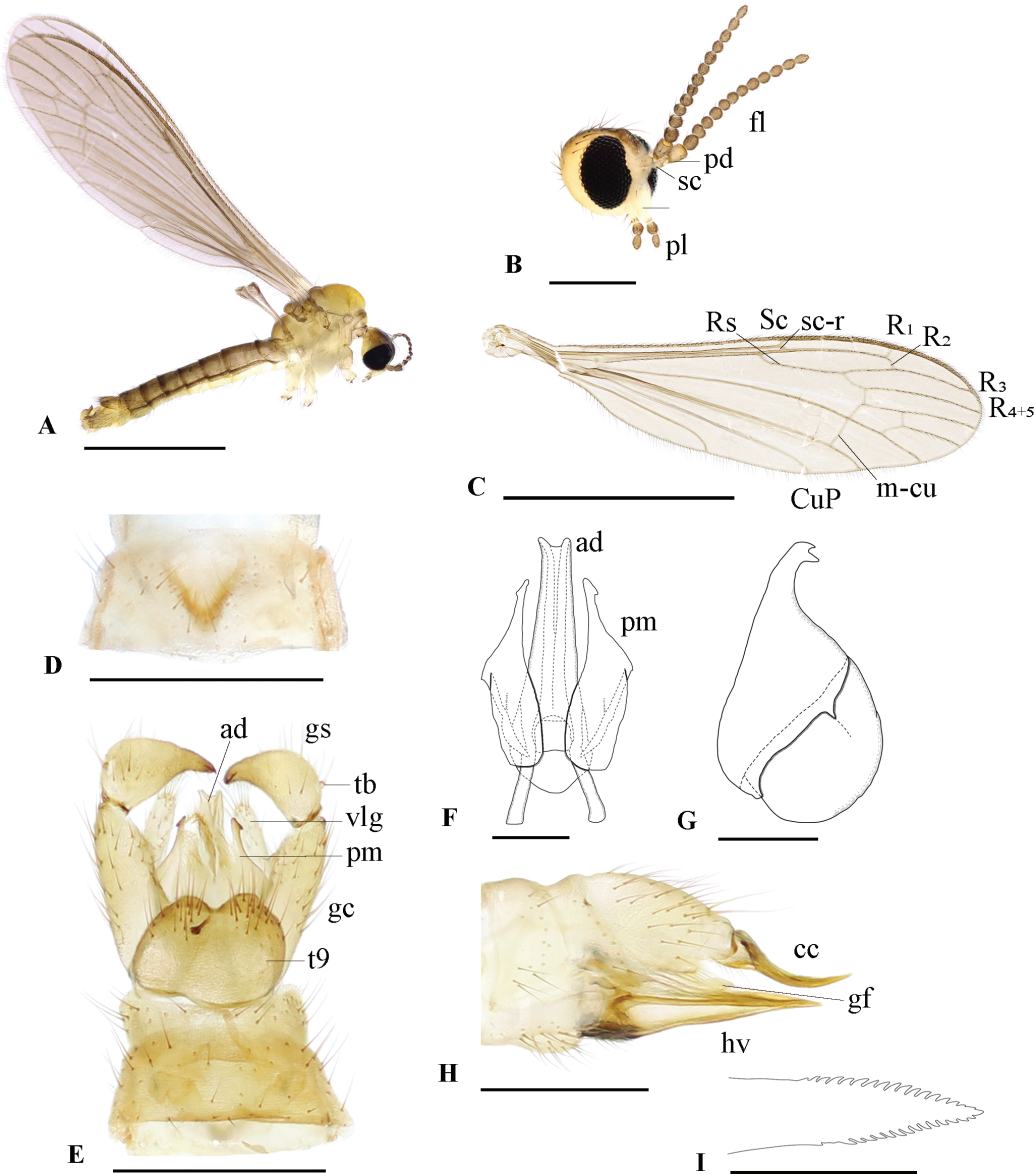
Dicranomyia (Erostrata) jejuensis sp. nov. **A** habitus, male (paratype) **B** head, female (paratype) **C** wing, male (paratype) **D** male seventh sternite, ventral view **E** male terminalia, dorsal view **F** aedeagal complex, dorsal view **G** paramere, lateral view **H** female terminalia, lateral view **I** tip of hypovalva, lateral view. Abbreviations: ad – aedeagus; cc – cercus; fl – flagellum; gc – gonocoxite; gf – genital fork; gs – gonostylus; hv – hypovalva; pd – pedicel; pl – palpus; pm – paramere; sc – scape; tb – tubercle; t9 – ninth tergite; vlg – ventromesal lobe of gonocoxite. Scale bars: 2 mm (**A, C**); 0.5 mm (**B, D, E, H**); 0.1 mm (**F, G, I**).

**Male (holotype).** Body length 4.3 mm, wing length 4.6 mm, antenna length 0.9 mm. General body coloration pale yellow to yellowish brown (Fig. 1A).

***Head*** (Fig. 1B). Dark brown dorsally, pale ventrally. Antennae 14-segmented; scape pale yellow; pedicel yellowish brown; flagellum brown. Rostrum pale, rudimentary. Palpus 3-segmented; basal 2/3 of first palpomere pale; remainder of palpus brown.

***Thorax*.** Prescutum and presutural scutum yellow. Postsutural scutum, scutellum and mediotergite yellowish brown. Pleuron uniformly dull yellow, without lateral stripes. Wing (Fig. 1C) tinged with pale brown; veins brown; Sc ending before middle of Rs; sc-r at tip of Sc; Rs arched at base; R_1_ and R_2_ nearly transverse, at the same level; R_3_ and R_4+5_ parallel to each other; discal medial cell closed; m-cu before fork of M; CuP ending beyond tip of Sc. Halter pale brown. Legs with coxae and trochanters pale; base of femora pale, remainder of femora brown; tibiae and tarsi brown. Femur II 3.3 mm; femur III 3.8 mm; tibia II 3.5 mm; tibia III 4.0 mm; tarsus II 3.2 mm; tarsus III 2.8 mm. Claw without additional tooth.

***Abdomen*.** Tergites yellowish brown, sternites 1–4 yellow, remaining yellowish brown. Seventh sternite with shallow V-shaped notch with darkened margin (Fig. 1D).

***Male terminalia*** (Fig. 1E–G). Yellow. Ninth tergite with posterior margin rounded (Fig. 1E), medially with distinct emargination, distal part covered with setae. Gonocoxite elongated, approximately 3× as long as width at base, with elongated, setose ventromesal lobe. Gonostylus yellowish brown at base, turning dark distally; distal 1/2 of gonostylus gradually tapered toward apex, with short black spine at tip; dorsal margin near the base with small tubercle bearing pale, stout seta. Paramere (Fig. 1F, G) with basal 1/2 pale and apical 1/2 yellowish, distal lobe darkened apically with hooked tip and subapical projection. Aedeagus (Fig. 1F) as long as gonocoxite, bifid, curved outwards at tip.

**Female.** Body length 4.5 mm, wing length 4.8 mm, antenna length 0.9 mm (*N* = 1). General body coloration brighter than male. Femur I 2.8 mm; II 3.2 mm; III 3.4 mm; tibia I 3.2 mm; I: 3.1 mm; III 3.4 mm; tarsus I 3.0 mm; II 2.6 mm; III 2.4 mm.

***Female terminalia*** (Fig. 1H–I). Yellow. Cercus curved upwardly (Fig. 1H), wider at base, narrowing towards acute tip. Genital fork long, ca 1.5× as long as width, extending to base of cercus. Hypovalva wedge-shaped, reaching to ca 2/3 of cercus, with distinct black spot at base. Dorsal and ventral margin of hypovalva serrated near tip (Fig. 1I).

###### Etymology.

Specific name “*jejuensis*” refers to the type locality, Jejudo Island, Korea.

###### Distribution.

The species is currently only known from Jejudo Island, Korea.

###### Habitats.

**Figure 2.**
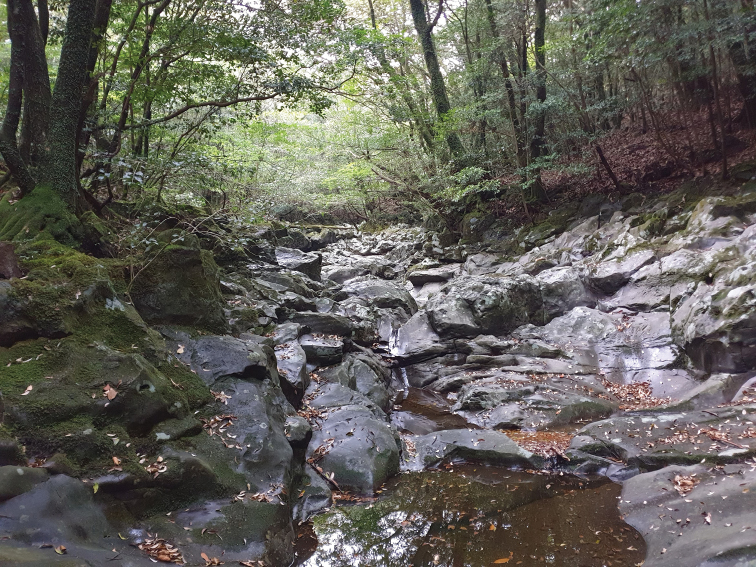
Habitat of Dicranomyia (Erostrata) jejuensis sp. nov.

Adults of this species are found in deciduous forests with moss-covered rocks along intermittent, rocky mountain streams (Fig. 2) and co-occur with D. (E.) submelas.

###### Period of activity.

Adults were collected from June through early September.

###### Remarks.

Dicranomyia (E.) jejuensis sp. nov. is morphologically similar to D. (E.) yazuensis based on the male genital structures, but it can be distinguished by the following characters: pleuron entirely dull yellow (vs dark dorsally); palpus 3-segmented (vs 2-segmented); distal 1/2 of gonostylus tapered to tip (vs distal 2/3 strongly narrowed toward tip); posterior margin of male seventh sternite with shallow V-shaped notch (vs long triangular notch); distal part of paramere with hooked tip (vs straight tip).

##### ﻿ Dicranomyia (Erostrata) koreana sp. nov.

Taxon classificationAnimaliaDipteraLimoniidae

9F0ADF72-CF20-5AA1-80E4-B4473CD6363C

https://zoobank.org/163BF75E-826C-4E64-AAE4-CFAA23F2E22D

Figs 3 , 4

###### Type material.

***Holotype***: Korea • ♂; Jeju-do, Seogwipo-si, Hawon-dong, Mt. Hallasan; 33°20.95'N, 126°29.72'E; alt. 1220 m; 13 Jun.–4 Aug. 2021; Y. J. Bae leg.; Malaise trap; GenBank: OM102979; CF21-0148; NIBR.

***Paratypes***: Korea • 1 ♂; Gyeonggi-do, Gapyeong-si, Buk-myeon, Jeokmok-ri, Garim-gyo (Br.); 37°58.60'N, 127°26.55'E; alt. 300 m; 25 Jul.–1 Aug. 2015; Y. J. Bae leg.; Malaise trap; published as D. (E.) tabashii by Podenas et al. (2019); KUEM • 2 ♂♂, 1 ♀; same data as for preceding; 2–8 Aug. 2015; published as D. (E.) tabashii by Podenas et al. (2019); KUEM • 1 ♂; same data as for preceding; 23–29 Jul. 2016; published as D. (E.) tabashii by Podenas et al. (2019); KUEM • 1 ♂; Gangwon-do, Inje-gun, Girin-myeon, Bangdong-ri, Mt. Bangtaesan; 37°54.50'N, 128°24.41'E; alt. 690 m; 30 Jul.–16 Sep. 2019; Y. J. Bae leg.; Malaise trap; KUEM • 1 ♂; Gyeonsangnam-do, Sancheong-gun, Sicheon-myeon, Jungsan-ri, Jungsan-ri Campsite, Mount Jirisan; 35°18.63'N, 127°45.09'E; alt. 700 m; 28 Jul. 2021; J. Kim, C. Lim, D. Lee, W. Lee leg.; sweeping; GenBank: OP081140; CF21-0152; KUEM.

###### Diagnosis.

Palpus is 3-segmented. Center of male seventh sternite has a deep conical internal sac that has a wide, round entrance. Outer face of gonostylus has two setae arising from a small tubercle. Paramere is elongated and narrow, distally with a darkened tip.

###### Description.

**Figure 3.**
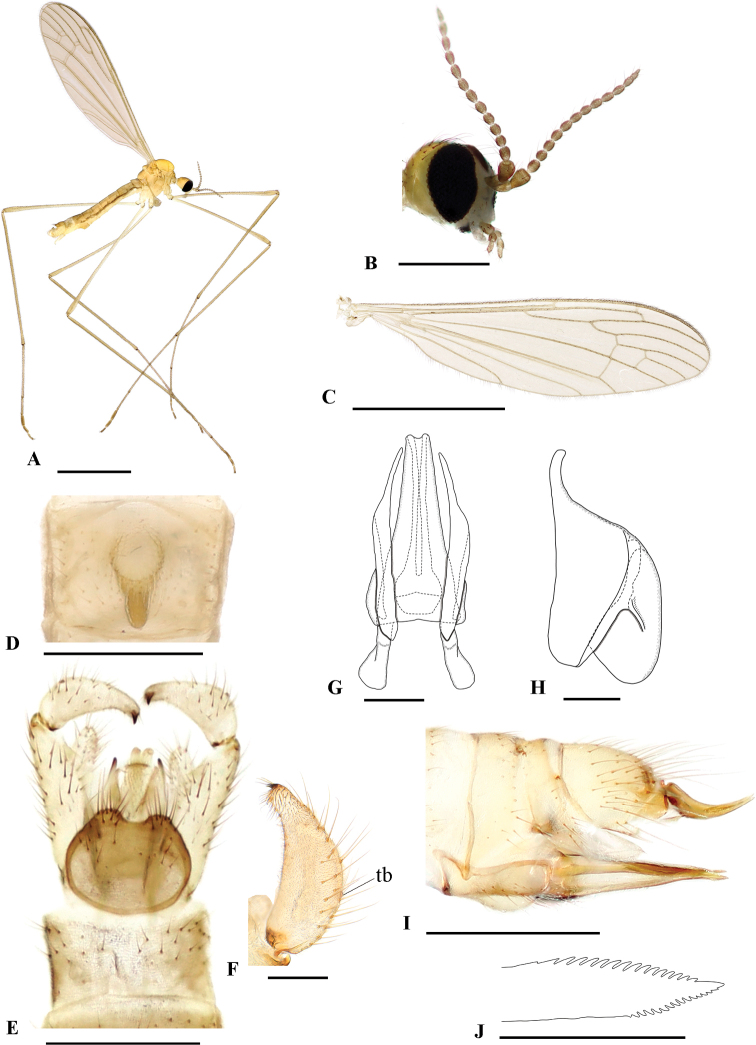
Dicranomyia (Erostrata) koreana sp. nov. **A** habitus, male (holotype) **B** head, male (paratype) **C** wing, male (paratype) **D** male seventh sternite, ventral view **E** male terminalia, dorsal view **F** gonostylus, ventral view **G** aedeagal complex, dorsal view **H** paramere, lateral view **I** female terminalia, lateral view **J** tip of hypovalva, lateral view. Abbreviation: tb – tubercle. Scale bars: 2 mm (**A, C**); 0.5 mm (**B, D, E, I**); 0.1 mm (**F, G, H, J**).

**Male (holotype).** Body length 3.5 mm, wing length 4.5 mm, antenna length 0.7 mm. General body coloration yellow (Fig. 3A).

***Head*** (Fig. 3B). Dark brown dorsally, yellow ventrally. Vertex with a distinct black spot between compound eyes. Antennae 14-segmented; scape pale brown; pedicel yellowish brown; flagellum brown. Rostrum pale, reduced. Palpus 3-segmented, yellowish brown; basal 1/2 of first palpomere pale.

***Thorax*.** Prescutum, scutum and scutellum yellow. Mediotergite yellowish brown. Pleuron entirely pale yellow, without lateral stripes. Wing (Fig. 3C) tinged with pale brown; veins brown; tip of Sc reaching ca 1/3 of Rs; sc-r at tip of Sc; R_1_ indistinct; R_2_ ending distinctly beyond tip of R_1_; discal cell closed; m-cu slightly beyond fork of M. Halter pale brown. Legs with coxae and trochanters pale yellow; femora and tibiae brownish yellow; tarsal segments light brown. Femur I 2.4 mm; femur II 2.8 mm; femur III 3.1 mm; tibia I 2.8 mm; tibia II 2.4 mm; tibia III 2.8 mm; tarsus I 2.9 mm; tarsus II 2.5 mm; tarsus III 2.4 mm. Claw without additional tooth.

***Abdomen*.** Tergites yellow except pale eighth tergite; sternites paler. Seventh sternite (Fig. 3D) with central deep, conical, slightly darkened sac, and rounded entrance.

***Male terminalia*** (Fig. 3E–H). Yellow. Ninth tergite rounded (Fig. 3E), wider basally, narrower apically; posterior margin with two short, setose lateral lobes separated by shallow U-shaped incision. Gonocoxite elongated, ventromesal lobe margin rounded and covered with setae, reaching beyond tip of aedeagus. Gonostylus (Fig. 3E, F) widened at base and narrowed at apex, with short, black dorsal spine at tip; dorsal margin near the base with small protuberance bearing two pale setae. Paramere (Fig. 3G, H) with basal part bilobed, distal lobe elongated and narrow, slightly darkened at tip. Aedeagus (Fig. 3G) apically bent downwards, bifid at tip.

**Female.** Body length 3.8 mm, wing length 4.7 mm, antenna length 0.7 mm (*N* = 1). General body coloration lighter than male.

***Female terminalia*** (Fig. 3I–J). Yellow. Cercus curved dorsally (Fig. 3I), gradually tapered to pointed tip. Genital fork broad, as long as width, not extending to base of cercus. Hypovalva elongated, blade-shaped reaching slightly before tip of cercus, with distinct dark spot at basal area. Distal end bearing dorsal and ventral serration (Fig. 3J).

###### Etymology.

Specific name “*koreana*” refers to the country of its discovery, Korea.

###### Distribution.

The species is widely distributed in Korea, including Jejudo Island.

###### Habitats.

**Figure 4.**
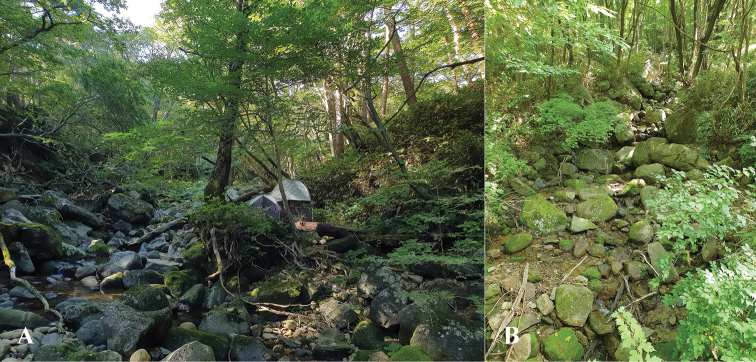
Habitats of Dicranomyia (Erostrata) koreana sp. nov. **A** Jejudo Island **B** Mt. Jirisan.

This species is found along intermittent mountain streams in moist mixed forests with grassy vegetation (Fig. 4A) and in wet deciduous forest along the rocky margins of small mountain streams (Fig. 4B). Adults share their habitats with D. (E.) globulithorax on Mount Bangtaesan and with D. (E.) tabashii on Mount Jirisan.

###### Period of activity.

Adults are mainly active from July through August.

###### Remarks.

In terms of the shape of the male terminalia, D. (E.) koreana sp. nov. is similar to D. (E.) tabashii, but it can be distinguished by the following characters: palpus 3-segmented (vs 2-segmented); male seventh sternite with weakly darkened, conical internal sac with round entrance (vs strongly darkened, U-shaped internal sac); paramere with darkened tip (vs without). This species is also similar to another species, D. (E.) jejuensis sp. nov. based on the male genital structures, but it can be distinguished by the following characters: male seventh sternite with a deep, conical internal sac (vs shallow, V-shaped notch); gonostylus with two setae from tubercle (vs a single seta); paramere without hook at tip (vs with hook).

The male genitalia of D. (E.) koreana sp. nov. from Mount Bangtaesan differs from other materials of the species based on the shape of the seventh sternite internal sack (shallow conical without rounded mouth) and paramere distal lobe (pointed tip). However, additional specimens are needed to determine whether this difference is due to intra- or interspecific variation.

### ﻿DNA barcode analysis

**Figure 5.**
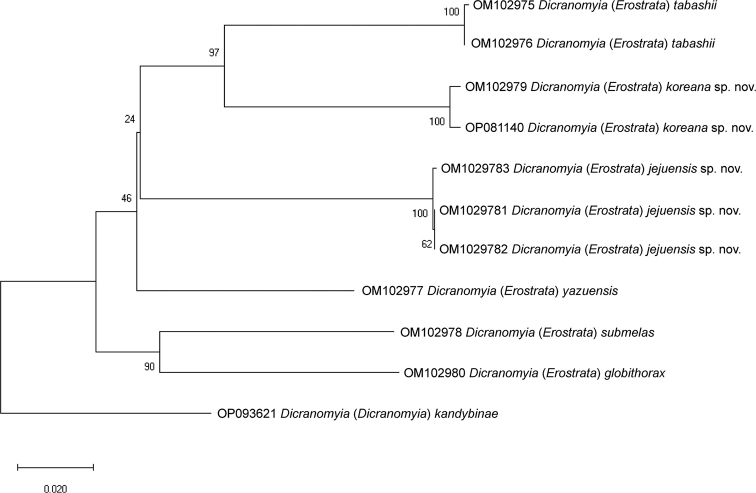
Neighbor-joining (NJ) Kimura-2-parameter tree based on the analysis of the *COI* of six Korean Dicranomyia (Erostrata) species and D. (Dicranomyia) kandybinae as outgroup. Numbers at the nodes indicate NJ bootstrap support values.

The 676-bp *COI* sequences contained 190 variable sites, of which 156 were parsimony-informative. The interspecific divergences (*p*-distances) within subgenus D. (Erostrata) ranged from 11.54% to 16.42%, with a mean distance of 13.17% across the entire dataset (Table 2), whereas the intraspecific genetic distances ranged from 0% to 0.59%: from 0% to 0.15% in D. (E.) jejuensis sp. nov., 0.59% in D. (E.) koreana sp. nov., and 0.15% in D. (E.) tabashii. The maximum intraspecific genetic distance (0.59%) was much smaller than the minimum interspecific one (11.54%). The NJ tree (Fig. 5) indicated that the monophyly of each of the new species was highly supported, as was that of subgenus D. (Erostrata) (Fig. 5).

**Table 2.** Estimates of genetic divergence (%) between sequences. The number of base differences per site from between sequences are shown. Standard errors (%) are shown above the diagonal and were obtained by a bootstrap procedure (1,000 replicates). All positions containing gaps and missing data were eliminated (complete delete option).

	Species	Accession number	1	2	3	4	5	6	7	8	9	10	11
1	D. (E.) globithorax	OM102980	–	1.36	1.36	1.36	1.36	1.37	1.28	1.42	1.41	1.37	1.34
2	D. (E.) jejuensis sp. nov.	OM102981	13.61	–	0	0.15	1.37	1.35	1.36	1.34	1.33	1.33	1.45
3		OM102982	13.61	0	–	0.15	1.37	1.35	1.36	1.34	1.33	1.33	1.45
4		OM102983	13.76	0.15	0.15	–	1.36	1.34	1.36	1.34	1.33	1.34	1.45
5	D. (E.) koreana sp. nov.	OM102979	15.24	14.94	14.94	14.79	–	0.30	1.38	1.21	1.20	1.31	1.45
6		OP081140	15.38	14.64	14.64	14.50	0.59	–	1.40	1.22	1.21	1.31	1.45
7	D. (E.) submelas	OM102978	11.54	14.64	14.64	14.79	15.09	15.68	–	1.43	1.43	1.37	1.36
8	D. (E.) tabashii	OM102975	16.12	14.94	14.94	14.79	11.69	11.83	16.42	–	0.15	1.29	1.44
9		OM102976	15.98	14.79	14.79	14.64	11.54	11.69	16.27	0.15	–	1.30	1.44
10	D. (E.) yazuensis	OM102977	15.24	12.43	12.43	12.57	13.46	13.46	13.76	12.87	13.02	–	1.30
11	D. (D.) kandybinae	OP093621	14.50	16.27	16.27	16.42	16.12	16.12	14.50	15.68	15.53	12.57	–

## ﻿Discussion

This is the first study to use DNA barcoding for the delimitation of the D. (Erostrata) species. The present study identified two new species using both morphological and molecular data. According to the NJ tree (Fig. 5), the subgenus includes two major clades, which can be distinguished based on the presence or absence of numerous black, strong spines on the mesal face of gonostylus. Indeed, D. (E.) jejuensis sp. nov., D. (E.) koreana sp. nov., D. (E.) tabashii, and D. (E.) yazuensis can be distinguished from other members of their subgenus based on the shape and mesal face (without lots of black, strong spines) of their gonostyli. Two hypotheses may be considered: i) this clade can be classified into morphological species groups, or ii) it can be elevated to a new subgenus. Additional materials are needed to more accurately reconstruct phylogenetic relationships within genus *Dicranomyia*.

Based on our morphological examinations of the materials, we also found that some specimens identified as D. (E.) tabashii by Podenas et al. (2019) are actually specimens of D. (E.) koreana sp. nov. Based on our observation, unknown cryptic species of crane flies could also be detected and identified using molecular data.

## Supplementary Material

XML Treatment for Erostrata

XML Treatment for Dicranomyia (Erostrata) jejuensis

XML Treatment for Dicranomyia (Erostrata) koreana
